# User Engagement and Feature Preferences in an AI-Powered mHealth Intervention for Diabetes Prevention: Secondary Analysis of a Randomized Controlled Trial

**DOI:** 10.2196/92981

**Published:** 2026-09-02

**Authors:** Benjamin Lalani, Gabriela Siew, Yllka Valdez, Aliyah Shehadeh, Daniel Zade, Kristin Riekert, Nestoras Mathioudakis, AI-DPP Study Group

**Affiliations:** 1 Harvard Medical School Harvard University Boston, MA United States; 2 Division of Endocrinology, Diabetes & Metabolism Department of Medicine Johns Hopkins University School of Medicine Baltimore, MD United States; 3 Division of Pulmonary and Critical Care Medicine Department of Medicine Johns Hopkins Medicine Baltimore, MD United States; 4 See Acknowledgments

**Keywords:** artificial intelligence, digital health, mobile app, prediabetes, obesity, overweight, diabetes prevention, lifestyle intervention, user engagement, reinforcement learning

## Abstract

**Background:**

Prediabetes is highly prevalent and increasing globally, yet lifestyle interventions remain underused. AI-driven mobile health (mHealth) tools can help scale diabetes prevention efforts, but the key factors driving their success are not well understood.

**Objective:**

This post hoc secondary analysis of a randomized controlled trial (RCT) aimed to characterize the most valued features and the role of user engagement in outcomes of a fully automated mHealth intervention for diabetes prevention.

**Methods:**

Data from 151 participants with prediabetes and overweight or obesity who were assigned to an AI-based diabetes prevention program (Sweetch) in a parent RCT (NCT05056376) were analyzed. Engagement (defined as the total number of days the app was used) was categorized into tertiles (low, medium, and high). Baseline characteristics were compared across engagement groups using ANOVA, Kruskal-Wallis, and chi-square tests, and regression models assessed the association between engagement and achievement of diabetes risk reduction outcomes (≥5% weight loss, ≥4% weight loss with ≥150 min/week of physical activity, or ≥0.2 percentage point reduction in hemoglobin A_1c_ [HbA_1c_] at 12 months). Perceived usefulness of intervention features was surveyed at 12 months.

**Results:**

Median engagement was 98 (IQR 34-232) days. Older age (*P*<.001) and lower baseline BMI (*P*=.04) were significantly associated with higher engagement. Compared with low engagement, high engagement was associated with greater odds of achieving the composite diabetes risk reduction outcome (odds ratio [OR] 2.59, 95% CI 1.11-6.01; *P*=.03), ≥5% weight loss (OR 3.31, 95% CI 1.16-9.42; *P*=.03), and ≥0.2 percentage point reduction in HbA_1c_ (OR 3.57, 95% CI 1.19-10.75; *P*=.02). Participants most frequently rated weight tracking, physical activity tracking, and the digital body weight scale as the features that were most helpful for achieving their health goals.

**Conclusions:**

Higher engagement with an AI-driven intervention requiring no human intervention was associated with improved diabetes risk reduction. Contrary to concerns about lower digital literacy, older adults engaged with the intervention more than younger adults. Features related to weight and physical activity tracking were most valued by patients in the program.

**Trial Registration:**

ClinicalTrials.gov NCT05056376; https://clinicaltrials.gov/study/NCT05056376

## Introduction

### Background

Mobile health (mHealth) technologies offer a promising modality for delivering diabetes prevention interventions at scale, with the potential to reach large populations at significantly lower costs than traditional, human-based interventions [1,2]. Various digital health tools, including mobile apps, web-based platforms, SMS text messaging, telemedicine, activity monitors, and voice calls, have been used to support diabetes prevention and weight management. These tools have yielded promising results in previous studies [3-5], and consequently, the Centers for Disease Control and Prevention (CDC) has recognized asynchronous, digital diabetes prevention programs (DPPs) within the National DPP framework [6].

Despite demonstrated efficacy in controlled settings, mHealth interventions have not translated into sustained real-world impact in diabetes prevention. Even with the availability of digital DPPs, which lower barriers to access compared with in-person programs, fewer than 1% of eligible adults participate in a DPP [7]. Engagement in intensive behavioral interventions is influenced by the perceived relevance, timing, and personalization of intervention content. With the emergence of AI, mHealth platforms have the potential to address these limitations by dynamically tailoring content delivery, feedback, and behavioral prompts to individual users at scale [8]. Unlike rule-based or static digital programs, AI-driven systems can adapt to user behavior over time, a capability that may enhance long-term engagement and increase participation. At the same time, skepticism persists regarding user willingness to engage with fully automated AI-based health interventions [9]. Concerns regarding trust and the perceived lack of human support raise important questions about whether AI-driven programs can sustain meaningful engagement over extended periods.

One such platform is Sweetch, an AI-driven, fully automated mHealth intervention that delivers personalized recommendations to promote physical activity and weight loss. A prior pilot study demonstrated the feasibility, acceptability, and short-term effectiveness of the Sweetch platform in adults with prediabetes [10]. A follow-up randomized, noninferiority clinical trial demonstrated that Sweetch was noninferior to the human coach–based DPP in reducing diabetes risk among patients with prediabetes [11]. However, beyond its clinical performance, the patterns, determinants, and perceived drivers of program engagement have not yet been examined. In addition, given the multicomponent nature of the Sweetch intervention, it remains unclear which features were perceived as most important in achieving these outcomes. Identifying these features may help inform the design and scaling of digital DPPs in the early stages of development [6].

### Study Objectives

We conducted a post hoc secondary analysis of a randomized controlled trial (RCT) to evaluate engagement with and perceived feature-specific usefulness of the AI-driven DPP over a 12-month period. The objectives of this study were to (1) characterize longitudinal engagement with an AI-driven DPP and identify participant characteristics associated with higher engagement, (2) evaluate the association between engagement and clinical outcomes, and (3) assess participants’ perceived usefulness of individual intervention features at the end of the intervention period. By elucidating how users interact with a fully automated AI-based DPP in real-world conditions, this study aims to inform the design, optimization, and scaling of future AI-driven diabetes prevention interventions.

## Methods

### Study Design and Population

The parent study was a multisite, pragmatic, parallel-group, 1:1 randomized noninferiority trial comparing a fully automated, AI-based DPP with a standard human-coached DPP over 12 months. Participants were randomized to either referral to an AI-powered DPP delivered through a mobile app and Bluetooth-enabled digital scale or referral to a CDC-recognized, human coach–led DPP delivered remotely over 12 months. The AI-led intervention provided personalized behavioral recommendations, educational content, self-monitoring tools, and adaptive push notifications without human coaching, whereas the comparator consisted of trained lifestyle coaches delivering the CDC PreventT2 curriculum through an initial core phase of 16 weekly sessions, followed by a maintenance phase of biweekly to monthly sessions. Both interventions were delivered independently of the study team after referral. The present study is a post hoc secondary analysis restricted to participants randomized to the AI-led DPP arm. The protocol and primary outcomes of the parent trial have been described elsewhere [11,12].

The parent trial was conducted from October 11, 2021, to December 16, 2024, with a planned 12-month follow-up for all participants. The 368 participants enrolled in the parent trial had laboratory-confirmed prediabetes and a race-specific BMI in the overweight or obese range (≥25 kg/m^2^ or ≥23 kg/m^2^ for Asian individuals). Exclusion criteria included a prior diagnosis of diabetes, severe cardiovascular conditions, factors affecting accuracy of hemoglobin A_1c_ (HbA_1c_), cognitive or psychiatric barriers to participation, and conditions or medications expected to substantially affect body weight or blood glucose.

In the parent trial, eligible participants were randomized in a 1:1 ratio to the AI-based or human coach–led DPP using centrally administered REDCap (Vanderbilt University) software. Randomization was stratified by recruitment site and baseline HbA_1c_ (≤6% vs 6.1%-6.4%) using randomly permuted block sizes of 2, 4, and 8. The allocation sequence was concealed from research coordinators responsible for participant enrollment and assignment until randomization occurred. Participants continued to receive routine medical care throughout the study. To minimize potential confounding, participation in other structured lifestyle interventions targeting nutrition, weight management, or diabetes prevention was not permitted, and medications expected to substantially influence body weight or glycemic control were prohibited during follow-up.

For this single-arm analysis, participants were included if they (1) were assigned to the AI arm of the trial and (2) had available outcomes data at 12 months. Participants were excluded from this analysis if they initiated any medications that could impact study outcomes during the intervention period, including antihyperglycemic agents, weight loss medications, or systemic steroids. No separate sample-size calculation was performed for this post hoc secondary analysis.

The clinical trial was conducted at 2 sites in the United States: Johns Hopkins Hospital, an urban tertiary academic medical center in Baltimore, Maryland; and Reading Hospital (Tower Health) in Reading, Pennsylvania, which serves a mixed urban, suburban, and rural population. The parent trial was prospectively registered at ClinicalTrials.gov (NCT05056376).

### Outcomes

The parent trial prespecified a primary composite outcome assessed at 12 months, defined as maintenance of HbA_1c_ <6.5% throughout follow-up and achievement of at least 1 of the following: (1) ≥5% weight loss, (2) ≥4% weight loss plus ≥150 minutes/week of moderate-to-vigorous physical activity (MVPA), or (3) an absolute HbA_1c_ reduction of ≥0.2 percentage points. Secondary end points were the individual components of the composite primary end point; attainment of CDC-recommended weekly physical activity levels at 12 months; and continuous changes in body weight, HbA_1c_, and average weekly physical activity over 12 months.

This secondary analysis focused on identifying factors associated with participant engagement and evaluating whether engagement level was associated with achieving end points related to weight loss, physical activity, and HbA_1c_ reduction. Thus, engagement served as the primary exposure variable, and the outcomes corresponded to the original end points from the parent trial.

Although the intervention incorporated Bluetooth-enabled devices and smartphone-derived data to support personalized recommendations, study outcomes were assessed independently. Body weight and HbA_1c_ were measured during study visits by trained research coordinators, and physical activity outcomes were derived from ActiGraph wrist monitors (Signant Health) worn for 7 consecutive days each month.

### Engagement Definition and Data Collection

Engagement definitions were not prespecified because engagement data were collected passively by the manufacturer’s platform, and the research team did not have access to these data until after trial completion. Participant engagement was automatically tracked by the Sweetch app (Sweetch Health Ltd) during months 1 to 11 of the trial and transferred to the research team after trial completion. Engagement was defined as the number of days over the course of the trial on which the Sweetch app was opened, and participants were categorized into tertiles based on cumulative use. Passive background processes (eg, automatic sensor data collection or data synchronization without opening the app) were not counted as engagement days. Engagement during month 12 was not included because participants completed their final study visits on different dates, resulting in unequal observation periods during the final month. Restricting engagement analyses to months 1 through 11 ensured a comparable exposure period for all participants before assessment of the 12-month outcomes. In addition, the number of days on which the app was opened in each month of the intervention period was assessed. Baseline characteristics were compared between engagement groups.

REDCap electronic data capture tools were used to collect participant baseline data and follow-up measures [12]. Baseline data were collected by trained research coordinators and included point-of-care HbA_1c_ measurements, height, weight, and demographics. Follow-up assessments at 6 and 12 months included repeat HbA_1c_ measurements, body weight, and surveys of perceived usefulness of intervention features. Physical activity was measured using ActiGraph wrist monitors worn for 7 consecutive days each month from randomization through month 11. MVPA was derived from raw accelerometer data using the Montoye wrist-worn cut point (≥3941 counts/min) [13]. Weekly MVPA was estimated as the mean across 11 monitoring periods.

### Intervention Description

The AI-based DPP used in this study was Sweetch. Participants received a health kit from Sweetch Health Ltd within 8 to 12 days after randomization. The kit included a Bluetooth-enabled digital body weight scale and instructions for app registration and synchronization. To simulate real-world conditions, research staff did not intervene in participants’ initiation or ongoing engagement with the app.

The intervention was fully automated, meaning that it was delivered without human coaching or scheduled human support after enrollment. Personalized recommendations and behavioral prompts were generated automatically by the AI-based platform, and use of the platform’s features (eg, tracking tools and the health education library) was driven solely by the participants themselves.

The manufacturer was permitted to implement updates to the intervention during the study period (refer to Multimedia Appendix 1 for details on the timing and content of these updates).

**Figure 1:**
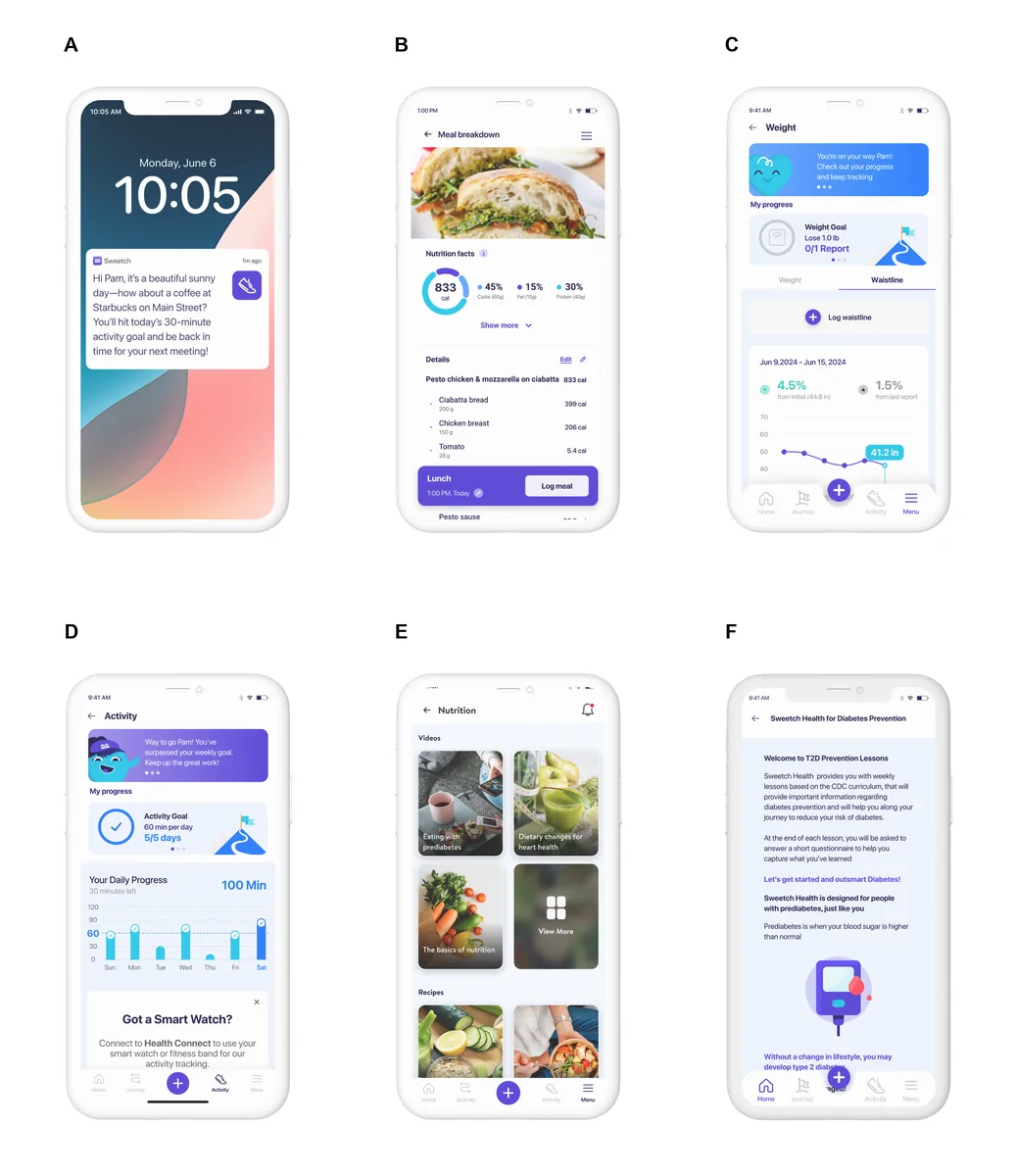
Features of the Sweetch intervention. (A) Just-in-time adaptive push notification, (B) meal-logging screen with a nutrition breakdown, (C) weight-tracking dashboard showing trend and goal progress, (D) physical activity module summarizing daily and weekly activity, (E) nutrition content library featuring videos and recipes, and (F) educational lesson on type 2 diabetes prevention.

The key features of the Sweetch intervention are displayed in Figure 1 and are summarized here.

Push notifications enable personalized, real-time nudges promoting adherence to physical activity, weight management, and dietary goals (Figure 1A). These notifications are generated based on both active data inputs (eg, body weight and meal logging) and passive data inputs (eg, geolocation, smartphone accelerometer data, and calendar) and use a reinforcement learning algorithm that iteratively adapts its recommendations by assigning rewards or penalties based on user behavior [14,15].

Nutrition tracking allows structured meal logging, including options for manual entry using a food library and automated photo-based meal detection. This feature replaced self-rated meal quality assessments in previous versions of the app (Figure 1B).

Weight tracking tracks weight trends over time through a Bluetooth-enabled digital scale, manual entries, or synchronization from third-party platforms. Visual graphs and logs allow users to monitor progress, while seamless integration with the Bluetooth-enabled scale ensures accurate, up-to-date data (Figure 1C).

Physical activity tracking automatically monitors users’ daily activities using smartphone accelerometers or integrated wearable devices (eg, Apple Health and Google Fit; Figure 1B). Users can also manually log activities such as Pilates, yoga, and swimming. The app provides insights into activity levels and sets adaptive, personalized goals to encourage gradual improvement (Figure 1D).

An educational content library provides evidence-based resources and lessons on type 2 diabetes (T2D) prevention, accessible within the app interface (Figures 1E and 1F)

“My Achievements” tracks user progress by displaying milestones and rewarding positive behaviors, offering visual motivation to continue healthy habits

“My Challenges” presents tailored health goals for users to complete, fostering resilience and a sense of accomplishment with each task achieved

“My Competitions” introduces friendly competition by allowing users to compare progress with peers, enhancing motivation and accountability through engaging contests

“My Leaderboard” displays user rankings based on activity and achievements, promoting motivation and community engagement through visible progress tracking

### Assessment of Perceived Feature Usefulness

After the intervention period (12 months), participants completed a survey assessing the perceived usefulness of specific app features in helping them achieve their health goals. The survey administered to participants is provided in Multimedia Appendix 1. Participants rated the following features on a 5-point Likert scale (1=“strongly disagree” to 5=“strongly agree”): push notifications, My Achievements, My Challenges, My Competitions, My Leaderboard, T2D prevention lessons, physical activity tracking, weight tracking, and the digital body weight scale. Four features were either discontinued before the trial (My Supporter and My Habits) or added during the intervention period (nutrition tracking and the educational content library) after survey finalization and were therefore not included in the feature usefulness assessment.

### Statistical Analysis

Baseline characteristics were summarized using medians and IQRs for continuous variables and frequencies with percentages for categorical variables. Differences in baseline characteristics across engagement categories (low, moderate, and high tertiles of total app use days) were assessed using 1-way ANOVA for normally distributed continuous variables, Kruskal-Wallis tests for nonnormally distributed continuous variables, and the Pearson chi-square tests for categorical variables. Monthly engagement patterns were examined descriptively across engagement groups.

To evaluate associations between engagement and study outcomes, logistic regression models were used for binary end points, including the primary composite end point and its individual components (eg, ≥5% weight loss, ≥0.2 percentage point reduction in HbA_1c_, and ≥4% weight loss combined with ≥150 minutes per week of physical activity), and linear regression models were used for continuous outcomes, including changes in body weight, HbA_1c_, and mean physical activity from baseline to 12 months. As an additional analysis, engagement was modeled as a continuous exposure, defined as total app-open days over the 330-day study period and scaled per 10-day increase for interpretability. For all analyses evaluating HbA_1c_ change at 12 months, models were restricted to participants with a baseline HbA_1c_ in the prediabetes range (5.7%-6.4%).

We performed a targeted sensitivity analysis adjusting for baseline BMI, baseline HbA_1c_, and baseline physical activity because these baseline measures correspond directly to the outcomes being evaluated (weight loss, HbA_1c_ reduction, and achievement of physical activity targets) and therefore could influence participants’ likelihood of achieving the study outcomes. Additional adjustment for age, sex, race, education, and study site was not performed because the limited number of outcome events would have resulted in low events-per-variable ratios and an increased risk of model overfitting and unstable coefficient estimates.

Missing data were handled using a complete-case approach. Participants without 12-month outcome data were excluded from the analytic cohort. Analyses of perceived feature usefulness included only participants who completed the 12-month follow-up survey. There was no missing engagement data for any participant, as these data were collected continuously and automatically. No imputation of missing data was performed for this secondary analysis.

All statistical tests were 2-tailed, and a significance threshold of *P*<.05 was used.

Due to the post hoc nature of the study, these analyses were exploratory, and the findings are presented as hypothesis generating.

### Ethical Considerations

The parent RCT (ClinicalTrials.gov; NCT05056376) was conducted in accordance with the Declaration of Helsinki and approved by the institutional review boards at Johns Hopkins Hospital and Reading Hospital (Tower Health). All participants provided written informed consent before randomization, and the original informed consent form and institutional review board approval permitted secondary analyses of the study data without additional participant contact or consent. This secondary analysis used deidentified data collected as part of the approved parent trial and did not require additional participant contact or consent. Participants received compensation for study-related assessments, as specified in the parent trial protocol [11,12]. No additional compensation was provided for this secondary analysis.

## Results

### Participants

**Figure 2:**
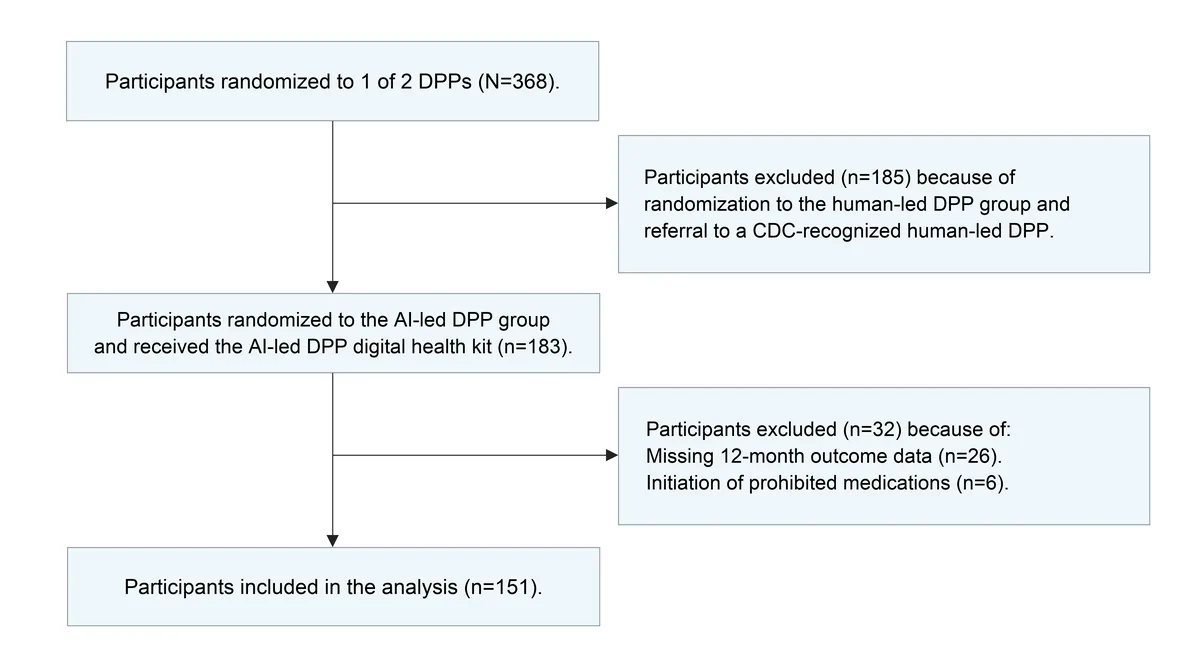
Study flow diagram. CDC: Centers for Disease Control and Prevention; DPP: diabetes prevention program.

Figure 2 illustrates participant flow from randomization through exclusions, resulting in 151 participants included in the final analytic sample; baseline characteristics are shown in Table 1. Participants were predominantly female and White, with high educational attainment and most being married or partnered. Recruitment occurred across 2 health systems. The median BMI was 32.3 (IQR 28.2-35.9) kg/m^2^, and the mean HbA_1c_ at baseline was 5.8% (SD 0.3%).

**Table 1:** Baseline characteristics of the study population (N=151).

Characteristics			Values
**Site, n (%)**			
	Johns Hopkins Hospital	93 (61.6)	
	Reading Hospital (Tower Health)	58 (38.4)	
Age (years), median (IQR)			61 (52-69)
**Sex, n (%)**			
	Female	96 (63.6)	
	Male	55 (36.4)	
**Race, n (%)**			
	Asian	10 (6.6)	
	Black or African American	35 (23.2)	
	White or Caucasian	99 (65.6)	
	>1 race	3 (2)	
	Other	4 (2.6)	
**Ethnicity, n (%)**			
	Not Hispanic or Latino	146 (96.7)	
	Hispanic or Latino	3 (2)	
	Declined to answer	1 (0.7)	
	Unknown	1 (0.7)	
**Marital status, n (%)**			
	Married or partnered	102 (67.5)	
	Previously married (divorced, separated, or widowed)	23 (15.2)	
	Single or other	26 (17.2)	
**Educational attainment, n (%)**			
	High school or less	22 (14.6)	
	Some college or associate degree	31 (20.5)	
	Bachelor degree	39 (25.8)	
	Graduate or professional degree	59 (39.1)	
BMI, median (IQR)			32.3 (28.2-35.9)
**BMI classification, n (%)**			
	Overweight	44 (29.1)	
	Class I obesity	59 (39.1)	
	Class II obesity	28 (18.5)	
	Class III obesity	20 (13.2)	
Hemoglobin A_1c_ (%), mean (SD)			5.8 (0.3)
Moderate-to-vigorous physical activity (min/week), median (IQR)			250 (112-404)
**Diet quality, n (%)**			
	Healthiest diet (1-5)	58 (38.4)	
	Moderately healthy diet (6-10)	42 (27.8)	
	Least healthy diet (11-16)	51 (33.8)	

### Engagement Patterns During the Intervention Period

**Figure 3:**
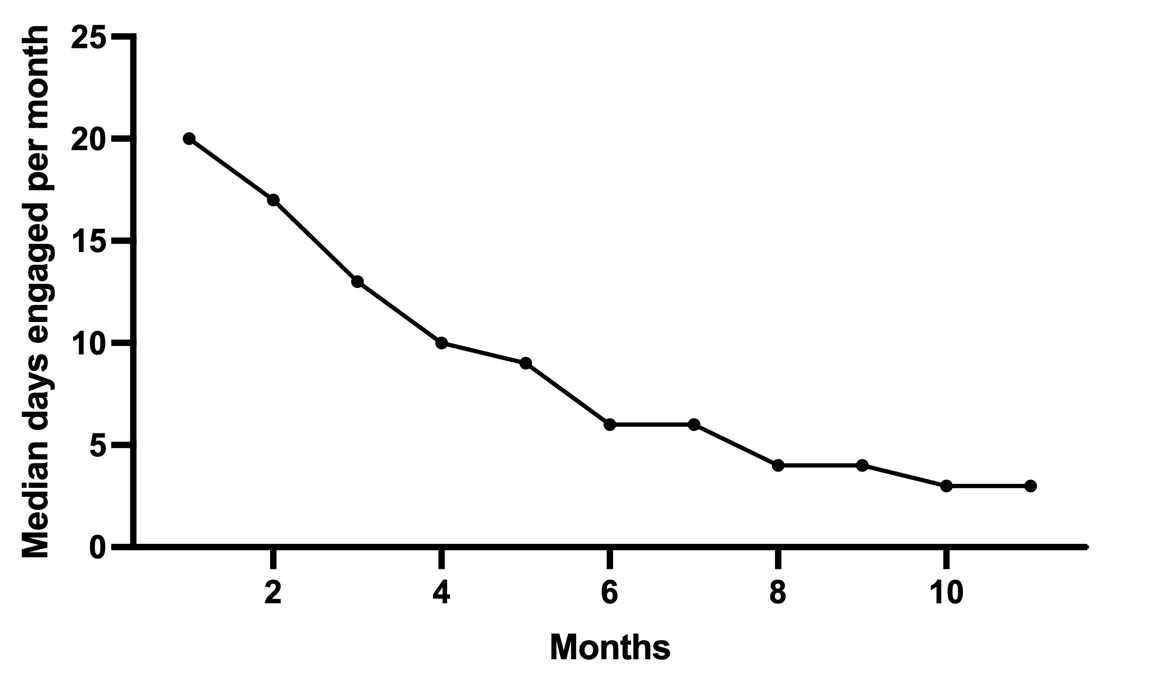
Engagement with the intervention during the study period. The median monthly engagement trajectory for all participants is shown over the intervention period.

Table 2 shows the distribution of total days engaged throughout the trial. Median engagement was 98 (IQR 34-232) days. The distribution was roughly bimodal, and engagement tertiles were defined as low (≤49 open days), moderate (50-175 open days), and high (>175 open days). The median number of days engaged per month among the cohort was 9 (IQR 3-21) days. Monthly engagement patterns (showing the median number of days engaged each month among the cohort) revealed a gradual decrease in app engagement over time (Figure 3).

**Table 2:** Distribution of total days engaged during the trial period (N=151).

Days engaged during trial period	Participants, n (%)
0	22 (14.6)
30	23 (15.2)
60	20 (13.2)
90	13 (8.6)
120	11 (7.3)
150	10 (6.6)
180	4 (2.6)
210	8 (5.3)
240	9 (6)
270	8 (5.3)
300	9 (6)
330	14 (9.3)

### Factors Associated With Increased Engagement

Several baseline factors were associated with engagement level. Participants in the low engagement group were significantly younger (median 52, IQR 42-63 years) than those in the moderate (median 63, IQR 55-68 years) and high (median 65, IQR 58-70 years) engagement groups (*P*<.001). They also had a higher median BMI (33.5, IQR 30.4-37.6 kg/m^2^) than those in the high engagement group (30.8 kg/m^2^, IQR 27.5-34.0; *P*=.04). There were no significant differences across engagement levels by sex, race, educational attainment, physical activity, or diet quality (Table S1 in Multimedia Appendix 1).

### Association Between Engagement and Outcomes

**Figure 4:**
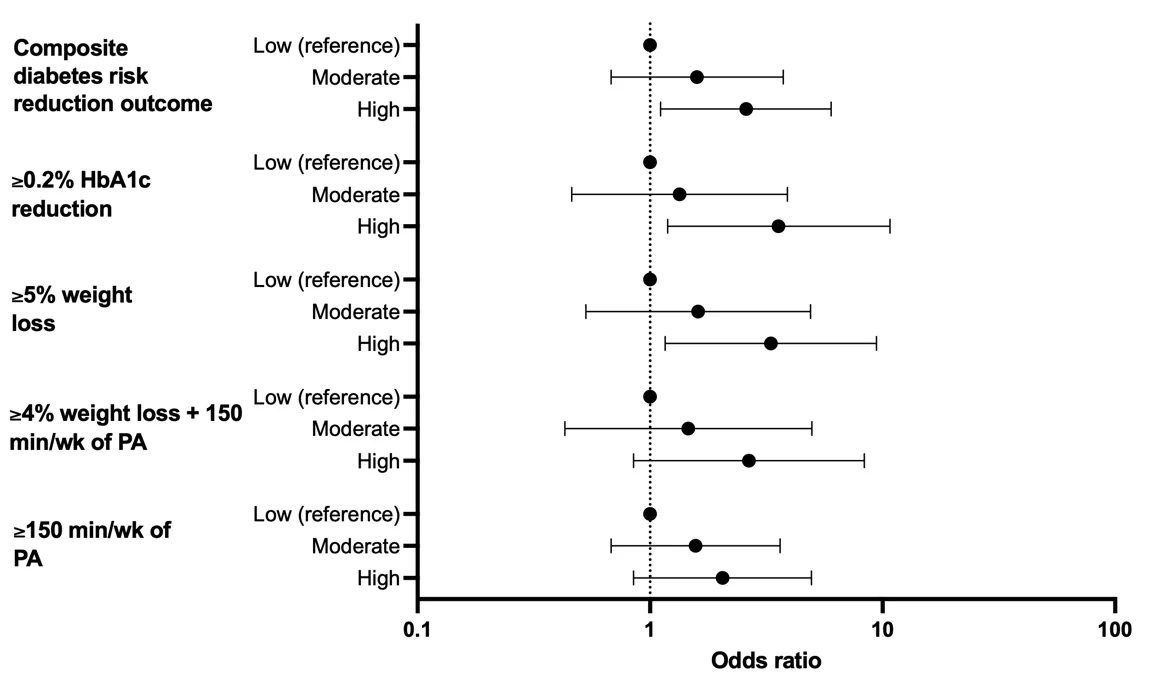
Odds ratios (ORs) for clinical outcomes at 12 months by engagement level. OR and 95% CI are shown for each engagement category, with low engagement as the reference group. Engagement categories represent tertiles based on the cumulative number of days the app was opened during the trial. HbA1c: hemoglobin A1c; PA: physical activity.

Higher engagement was associated with greater odds of achieving the composite diabetes risk reduction benchmark (odds ratio [OR] 2.59, 95% CI 1.11-6.01; *P*=.03), ≥5% weight loss (OR 3.31, 95% CI 1.16-9.42; *P*=.03), and a ≥0.2 percentage point reduction in HbA_1c_ (OR 3.57, 95% CI 1.19-10.75; *P*=.02) compared with low engagement (Figure 4). Moderate engagement was not significantly associated with any outcome. Engagement level was not significantly associated with achieving ≥4% weight loss combined with 150 minutes per week of physical activity or with meeting the physical activity threshold alone. The sensitivity analysis of the primary composite outcome, adjusting for baseline BMI, baseline HbA_1c_, and baseline physical activity, yielded results consistent with the primary analysis (Table S2 in Multimedia Appendix 1).

In linear regression analyses of continuous outcomes, high engagement was associated with greater weight loss at 12 months, whereas no significant associations were observed for changes in HbA_1c_ or physical activity (Table S3 in Multimedia Appendix 1). When engagement was modeled as a continuous variable, higher engagement was associated with modest increases in the odds of achieving the composite diabetes risk reduction outcome, ≥0.2 percentage point reduction in HbA_1c_, and ≥5% weight loss, with no significant associations observed for physical activity outcomes (Table S4 in Multimedia Appendix 1).

### User Preferences After 12 Months of Intervention

**Figure 5:**
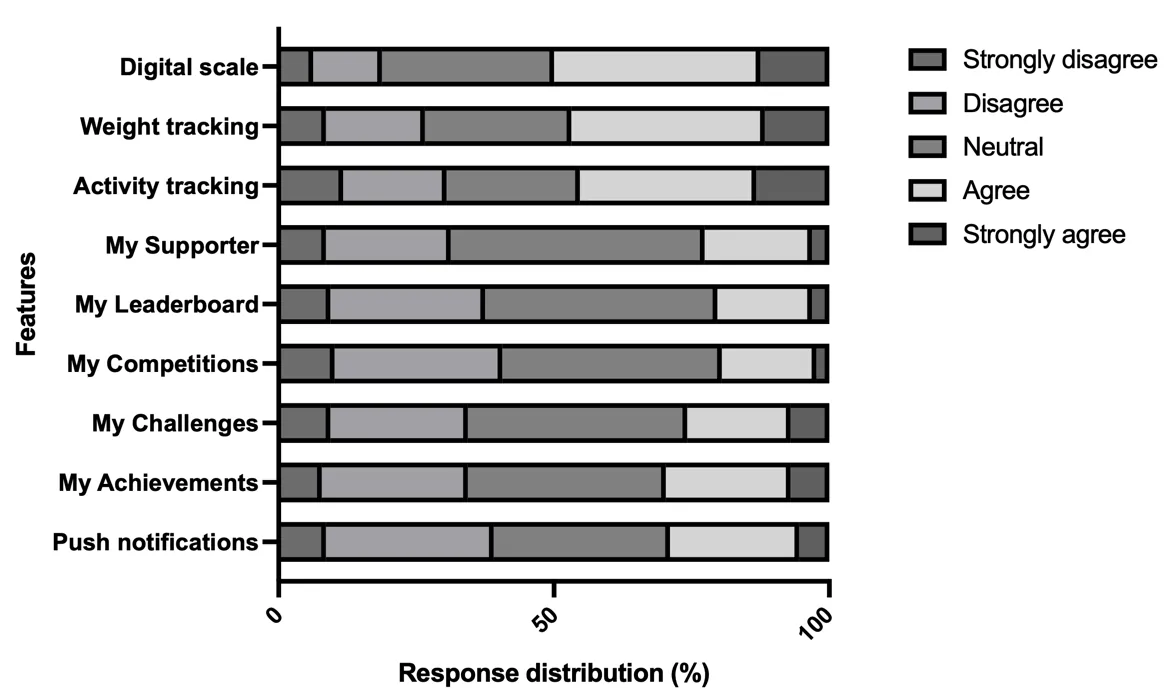
Perceived usefulness of features in the intervention. The horizontal stacked bar chart shows the percentage distribution of responses to survey items assessing whether each app feature helped participants achieve their goals. Responses were recorded on a 5-point Likert scale ranging from “strongly disagree” to “strongly agree.” Each bar represents 1 feature and sums to 100% of responses (n=128 per item).

Survey data were available for 128 of 151 (84.8%) participants included in the analytic sample. Participants’ perceptions of whether individual app features helped them achieve their goals are summarized in Figure 5, with corresponding raw counts and percentages reported in Table S5 in Multimedia Appendix 1. For several features, a neutral response was the most frequently selected among the 128 participants, including achievements (n=46, 35.9%), challenges (n=51, 39.8%), competitions (n=51, 39.8%), leaderboard (n=54, 42.2%), and T2D prevention lessons (n=59, 46.1%).

In contrast, tracking-oriented features elicited more favorable perceptions. For activity tracking, 58 (45.3%) participants reported agreeing or strongly agreeing, compared with 39 (30.5%) reporting disagreeing or strongly disagreeing. Similar patterns were observed for weight tracking, with 60 (46.9%) participants reporting agreeing or strongly agreeing. The digital scale demonstrated the most positive response distribution overall, with 64 (50%) participants agreeing or strongly agreeing.

For push notifications, among the 128 participants, 39 (30.5%) indicated disagreement, 41 (32%) were neutral, 30 (23.4%) indicated agreement, and 7 (5.5%) indicated strong agreement.

## Discussion

### Principal Findings

In this secondary analysis of an RCT, engagement (ie, greater amounts of app use) with an AI-led DPP was associated with achieving the CDC’s diabetes risk reduction benchmark and recommended thresholds for weight loss and HbA_1c_ reduction. These findings reinforce app engagement as an important mechanism through which mHealth interventions may exert clinical benefit and extend prior digital health literature by demonstrating this relationship in the context of a fully automated AI-based program delivered over a year-long intervention period [3,16-18].

### Factors Associated With Engagement

Engagement varied by participant characteristics. Older participants demonstrated higher levels of engagement, challenging assumptions that older adults engage less with digital health interventions [19,20]. This may reflect greater health motivation, more available time, or a stronger perceived need among older users. Participants with higher baseline BMI engaged less with the intervention, which may be a result of greater physical, behavioral, or psychological barriers to sustained self-monitoring and physical activity. Because participants with higher baseline BMI are at greater metabolic risk, lower engagement in this subgroup raises the possibility that additional tailoring, support, or re-engagement strategies may be needed to ensure that fully automated DPPs do not inadvertently widen disparities by preferentially benefiting individuals at lower metabolic risk.

Engagement with the intervention declined over time, which may reflect a natural waning of novelty, behavior change fatigue, and/or a perceived decline in the app’s ongoing value. Alternatively, it may indicate that users acquired the knowledge or behavioral reinforcement they needed early on, reducing their reliance on the app over time. The Hawthorne effect, whereby initial behavior changes are driven by awareness of being observed, may also explain early spikes followed by tapering engagement [21]. The observed decline in engagement highlights the need for future work to identify strategies to maintain or re-engage users over time, particularly in longer-term prevention programs.

### Interpretation of the Engagement-Outcome Association

This exploratory analysis identified an association between higher engagement with the intervention and a greater likelihood of achieving diabetes risk reduction outcomes. However, several alternative explanations should be considered. Participants who engaged with the app more frequently may have differed systematically from those with lower engagement in ways not captured by measured baseline characteristics, including motivation, self-regulation, or readiness for behavior change. Reverse causality is also possible, as participants who experienced early weight loss or other positive reinforcement may have become more engaged over time rather than engagement itself driving these improvements. Consequently, the observed associations should not be interpreted as causal, and residual confounding cannot be excluded.

Notably, the engagement metric used in this analysis may not fully capture all aspects of intervention engagement. Some components of the Sweetch intervention, including meal logging, weight tracking, educational content, and progress monitoring, require active interaction with the app. In contrast, the AI-generated push notifications were designed to deliver personalized behavioral support passively and could influence participants without requiring the app to be opened. Furthermore, the lifestyle behaviors targeted by the intervention occur outside the app, meaning that behavior change initiated through either active or passive intervention components may be sustained without ongoing app interaction.

### Perceived Usefulness of Features

Weight tracking, physical activity tracking, and use of the digital scale were more frequently endorsed as helpful in achieving health goals than social or gamified features, such as leaderboards and competitions, which were often rated as neutral. This aligns with findings from a large-scale evaluation of the NHS digital DPP, which found that participants most frequently engaged with tracking features, while peer support was rarely used [22].

Push notifications, which serve as the primary application for AI-driven personalization and are intended to approximate aspects of human coaching, were not perceived as among the most useful features, whereas participants more frequently endorsed self-monitoring features that do not rely directly on AI (eg, weight tracking, physical activity tracking, and the connected digital scale). These findings suggest that the contribution of AI-driven personalization to user engagement and clinical benefit remains uncertain and highlight the need for future studies to better define the most effective role of AI within digital DPPs.

In the context of the parent trial’s demonstration of noninferiority to a human-coached DPP, these findings raise the possibility that comparable clinical benefit may have been achieved through mechanisms distinct from human support, with advanced, low-burden self-monitoring capabilities playing a central role. Other programs, such as Lark [23], have similarly adopted AI-based personalization through chatbot interfaces, reflecting a broader trend toward individualized digital experiences. However, the relative value of these features remains uncertain from the user perspective. As digital DPPs continue to scale, the findings from this study underscore the need to better understand how users interact with individual components and suggest that refining core tracking functionality may be as important as, or potentially more impactful than, expanding complex personalization systems.

### Strengths

This study has strengths. It draws on prospectively collected data from a RCT with a 12-month follow-up period and high retention. Unlike prior studies that report engagement descriptively without treating it as a formal research end point, this study prospectively measured engagement, analyzed its association with clinical outcomes, and examined use patterns over time [24]. The pragmatic nature of the trial, specifically the absence of prompts from research staff to engage with the intervention, allowed participant behavior to more closely reflect real-world dynamics. The multisite design and diverse participant demographics enhance generalizability, and the use of validated, policy-relevant outcomes for diabetes risk reduction and objectively measured physical activity data strengthen the reliability of the findings.

### Limitations

Our study has limitations. Because the research team did not have access to the engagement data structure until after trial completion, engagement definitions and analytic thresholds could not be prespecified. Consequently, this post hoc secondary analysis should be considered exploratory and hypothesis generating, and the observed associations warrant confirmation in prospectively designed studies. Residual confounding by measured baseline factors (eg, sex, age, race, and education) and unmeasured baseline characteristics (eg, health status, motivation, and readiness for behavior change) cannot be excluded. Given the limited number of outcome events, adjusted multivariable models were not constructed because of potential overfitting and unstable coefficient estimates. Engagement was defined solely by the number of days the app was opened, which may not capture the quality or type of user interaction, although this is a commonly used metric for measuring engagement in mHealth interventions [24]. Engagement was categorized using sample-dependent tertiles due to the lack of accepted criteria for thresholds of meaningful or adequate use, limiting interpretability and comparability across studies. Consequently, the definitions of low, moderate, and high engagement used in this study may not generalize to other digital interventions or real-world populations. In addition, feature usefulness was assessed only at the 12-month follow-up and may therefore not reflect the perspectives of participants who disengaged previously and did not complete the survey. Iterative updates to the Sweetch platform during the study period could have influenced user experience, engagement patterns, and participants’ perceptions of individual intervention features, as not all participants were necessarily exposed to identical versions of the app throughout the study. Finally, the trial was conducted during the COVID-19 pandemic, which may have affected participants’ health behaviors or ability to engage with the app.

### Conclusions

In summary, this study found that higher engagement with a fully automated, AI-driven DPP was associated with clinically meaningful diabetes risk reduction outcomes. Engagement was notably higher among older adults and individuals with lower BMI, challenging assumptions about digital literacy barriers and suggesting possible differences in motivation or perceived need. Among the app’s features, integrated tracking tools, such as weight and activity monitoring, were rated most positively. Future research should further investigate the relative value of specific features within digital DPPs, particularly in fully automated platforms, and explore how such programs perform in broader real-world settings beyond the trial environment. As digital health interventions continue to expand, identifying the most effective and user-preferred components will be essential for designing scalable, cost-effective, and sustainable tools for diabetes prevention.

## Data Availability

As of the time of this submission, the authors do not have institutional review board (IRB) approval to share this dataset. However, the consent language used in the study may permit the data to be submitted to a controlled-access repository (eg, Vivli) following deidentification and IRB approval of a change in research.

## Appendix

Multimedia Appendix 1 Survey materials, software update details, baseline characteristics stratified by engagement level, baseline characteristics stratified by engagement levels, regression analyses examining associations between engagement and primary, continuous, and composite trial outcomes, and participant feature preference survey results.

Multimedia Appendix 2 CONSORT checklist.

## Abbreviations

CDC: Centers for Disease Control and Prevention
DPP: diabetes prevention program
HbA1c: hemoglobin A1c
mHealth: mobile health
MVPA: moderate-to-vigorous physical activity
OR: odds ratio
RCT: randomized controlled trial
T2D: type 2 diabetes
