# Supplementary material for: User Engagement and Feature Preferences in an AI-Powered mHealth Intervention for Diabetes Prevention: Secondary Analysis of a Randomized Controlled Trial
Source: JMIR Mhealth Uhealth. 2026 Sep 2;14:e92981. doi: 10.2196/92981 (PMC13583526; doi:10.2196/92981)
Supplement: Supplementary file 2 [file mhealth_v14i1e92981_app1.docx]

Supplementary Appendix

Contents

[Sweetch Features Questionnaire	2](#_Toc234240869)

[Platform Updates During the Study Period	4](#_Toc234240870)

[Table S1 Baseline Characteristics by Engagement Level	5](#_Toc234240871)

[Table S2: Analysis of the Primary Composite Outcome Adjusted for Baseline BMI, Baseline HbA1c, and Baseline Physical Activity	7](#_Toc234240872)

[Table S3: Association Between Engagement Levels and Continuous Outcomes	8](#_Toc234240873)

[Table S4: Association between Engagement (Continuous Outcome) and Study Outcomes	9](#_Toc234240874)

[Table S5: Feature Preferences from Survey Analysis	10](#_Toc234240875)

# Sweetch Features Questionnaire

# Platform Updates During the Study Period

The manufacturer was permitted to implement updates to the app during the trial. The Sweetch platform underwent multiple software updates during the study period. The initial version (Sweetch 1.0) was available at the start of the trial in October 2021. Subsequent updates included version 11.100.101 (August 2022), which introduced Sweetch 2.0 with redesigned home, nutrition, physical activity, and weight screens; version 11.100.106 (March 2023), which moved educational content to the home page; version 11.100.110 (April 2023), which redesigned location-based notifications; version 11.100.126 (May 2023), which added wearable-based physical activity tracking support; version 11.100.129 (July 2023), which redesigned physical activity notifications; version 11.100.130 (August 2023), which expanded the educational content library; version 11.100.131 (September 2023), which redesigned the home page to display daily goal achievements; and version 122.100.407 (December 2024), which redesigned the nutrition domain.

# Table S1 Baseline Characteristics by Engagement Level

| **Factor** | **Level** | **Low (N = 51)** | **Moderate (N = 51)** | **High (N = 49)** | **P value** |
| --- | --- | --- | --- | --- | --- |
| Site | Johns Hopkins Hospital | 35 (69%) | 30 (59%) | 28 (57%) | .44 |
|  | Reading Tower Health | 16 (31%) | 21 (41%) | 21 (43%) |  |
| Age, median (IQR) |  | 52.0 (42.0, 63.0) | 63.0 (55.0, 68.0) | 65.0 (58.0, 70.0) | <.001 |
| Sex | Female | 32 (63%) | 37 (73%) | 27 (55%) | .19 |
|  | Male | 19 (37%) | 14 (27%) | 22 (45%) |  |
| Race | Asian | 6 (12%) | 3 (6%) | 1 (2%) | .41 |
|  | Black or African American | 14 (27%) | 11 (22%) | 10 (20%) |  |
|  | White or Caucasian | 28 (55%) | 34 (67%) | 37 (76%) |  |
|  | More than One Race | 1 (2%) | 2 (4%) | 0 (0%) |  |
|  | Other | 2 (4%) | 1 (2%) | 1 (2%) |  |
| Ethnicity | Not Hispanic or Latino | 48 (94%) | 51 (100%) | 47 (96%) | .41 |
|  | Hispanic or Latino | 2 (4%) | 0 (0%) | 1 (2%) |  |
|  | Declined to Answer | 1 (2%) | 0 (0%) | 0 (0%) |  |
|  | Unknown | 0 (0%) | 0 (0%) | 1 (2%) |  |
| Marital Status | Married/Partnered | 30 (59%) | 37 (73%) | 35 (71%) | .59 |
|  | Previously Married (Divorced/Separated/Widowed) | 10 (20%) | 7 (14%) | 6 (12%) |  |
|  | Single/Other | 11 (22%) | 7 (14%) | 8 (16%) |  |
| Educational Attainment | High School or Less | 6 (12%) | 11 (22%) | 5 (10%) | .51 |
|  | Some College/Associate's Degree | 14 (27%) | 8 (16%) | 9 (18%) |  |
|  | Bachelor's Degree | 12 (24%) | 12 (24%) | 15 (31%) |  |
|  | Graduate/Professional Degree | 19 (37%) | 20 (39%) | 20 (41%) |  |
| Body Mass Index (BMI) , median (IQR) |  | 33.5 (30.4, 37.6) | 32.5 (28.8, 37.4) | 30.8 (27.5, 34.0) | .04 |
| BMI Classification | Overweight | 9 (18%) | 14 (27%) | 21 (43%) | .17 |
|  | Class I Obesity | 22 (43%) | 19 (37%) | 18 (37%) |  |
|  | Class II Obesity | 12 (24%) | 10 (20%) | 6 (12%) |  |
|  | Class III Obesity | 8 (16%) | 8 (16%) | 4 (8%) |  |
| Hemoglobin HbA1c Result (%), mean (SD) |  | 5.8 (0.3) | 5.9 (0.3) | 5.8 (0.3) | .05 |
| MVPA, min/week, median (IQR) |  | 269.0 (112.0, 430.0) | 221.0 (139.0, 407.0) | 268.0 (112.0, 363.0) | .83 |
| Diet Quality | Healthiest Diet (1-5) | 18 (35%) | 20 (39%) | 20 (41%) | .35 |
|  | Moderately Healthy Diet (6-10) | 15 (29%) | 10 (20%) | 17 (35%) |  |
|  | Least Healthy Diet (11-16) | 18 (35%) | 21 (41%) | 12 (24%) |  |

# Table S2: Analysis of the Primary Composite Outcome Adjusted for Baseline BMI, Baseline HbA1c, and Baseline Physical Activity

| **Variable** | **Adjusted OR^a^** | **95% CI** | **P value** |
| --- | --- | --- | --- |
| **Engagement tertile** |  |  |  |
| Low | Reference | Reference | Reference |
| Moderate | 1.43 | 0.60 to 3.44 | .42 |
| High | 2.88 | 1.19 to 6.97 | .02 |
| Baseline BMI | 1.01 | 0.96 to 1.07 | .63 |
| Baseline HbA1c | 3.21 | 0.88 to 11.75 | .08 |
| Baseline physical activity (per minute/week) | 0.999 | 0.997 to 1.000 | .08 |
| ^a^Odds ratios were estimated using multivariable logistic regression adjusted for baseline BMI, baseline HbA1c, and baseline physical activity. Low engagement served as the reference category. BMI = body mass index; HbA1c = hemoglobin HbA1c; OR = odds ratio; CI = confidence interval. | | | |

# Table S3: Association Between Engagement Levels and Continuous Outcomes

| **Outcome** | **Engagement Level** | **Result** |
| --- | --- | --- |
| **Weight Change at 12 months (%)** | Low | Reference |
|  | Moderate | Coef: 0.03 (-1.84–1.91), P = .97 |
|  | High | **Coef: -2.45 (-4.34–-0.55), P = .01** |
| **A1C Change at 12 months (%)** | Low | Reference |
|  | Moderate | Coef: 0.06 (-0.07–0.19), P = .33 |
|  | High | Coef: -0.09 (-0.23–0.05), P = .22 |
| **Average Physical Activity at 12 Months (min)** | Low | Reference |
|  | Moderate | Coef: 11.31 (-57.65–80.27), P = .75 |
|  | High | Coef: 61.64 (-8.02–131.30), P = .08 |

# Table S4: Association between Engagement (Continuous Outcome) and Study Outcomes

*Engagement modeled continuously (per 10 additional app-open days)*

| **Outcome** | **N** | **Odds Ratio (95% CI) per 10 Days^a^** | **P value** |
| --- | --- | --- | --- |
| Composite Diabetes Risk Reduction Benchmark | 151 | 1.03 (1.00–1.06) | .05 |
| ≥0.2% HbA1c Reduction^b^ | 106 | 1.04 (1.00–1.08) | .04 |
| ≥4% Weight Loss + 150 Min/Week of PA | 151 | 1.04 (1.00–1.08) | .07 |
| ≥5% Weight Loss | 151 | 1.04 (1.00–1.07) | .05 |
| ≥150 min/week physical activity | 151 | 1.02 (0.99–1.06) | .15 |
| ^a^Odds ratios were estimated using unadjusted logistic regression models with engagement modeled as a continuous variable, scaled per 10 additional app-open days. ^b^Analysis restricted to participants eligible for the HbA1c outcome. | | | |

# Table S5: Feature Preferences from Survey Analysis

| **Survey Item** | **Strongly Disagree  n (%)** | **Disagree  n (%)** | **Neutral  n (%)** | **Agree  n (%)** | **Strongly Agree  n (%)** |
| --- | --- | --- | --- | --- | --- |
| **Push notifications** helped me achieve my goals | 11 (8.6) | 39 (30.5) | 41 (32.0) | 30 (23.4) | 7 (5.5) |
| **My Achievements** feature helped me achieve my goals | 10 (7.8) | 34 (26.6) | 46 (35.9) | 29 (22.7) | 9 (7.0) |
| **My Challenges** feature helped me achieve my goals | 12 (9.4) | 32 (25.0) | 51 (39.8) | 24 (18.8) | 9 (7.0) |
| **My Competitions** feature helped me achieve my goals | 13 (10.2) | 39 (30.5) | 51 (39.8) | 22 (17.2) | 3 (2.3) |
| **My Leaderboard** feature helped me achieve my goals | 12 (9.4) | 36 (28.1) | 54 (42.2) | 22 (17.2) | 4 (3.1) |
| **T2D Prevention Lessons** helped me achieve my goals | 11 (8.6) | 29 (22.7) | 59 (46.1) | 25 (19.5) | 4 (3.1) |
| **Activity tracking** helped me achieve my goals | 15 (11.7) | 24 (18.8) | 31 (24.2) | 41 (32.0) | 17 (13.3) |
| **Weight tracking** helped me achieve my goals | 11 (8.6) | 23 (18.0) | 34 (26.6) | 45 (35.2) | 15 (11.7) |
| **Digital body weight scale** helped me achieve my goals | 8 (6.2) | 16 (12.5) | 40 (31.2) | 48 (37.5) | 16 (12.5) |
